# Comparison of Therapeutic Effects of PVP and PKP Combined With Triple Medication on Mild and Moderate Osteoporotic Vertebral Compression Fracture in the Elderly

**DOI:** 10.3389/fsurg.2021.663099

**Published:** 2022-03-25

**Authors:** Yi Zhou, Jiang Jiang, Fulong Gu, Daguo Mi

**Affiliations:** Department of Orthopedics and Traumatology, Nantong Hospital of Traditional Chinese Medicine, Affiliated Traditional Chinese Medicine Hospital of Nantong University, Nantong, China

**Keywords:** triple therapy, osteoporotic vertebral compression fracture, PVP, PKP, Oswestry dysfunction index

## Abstract

**Objective:**

To compare and analyze the therapeutic effect of percutaneous vertebroplasty (PVP) and percutaneous kyphoplasty (PKP) combined with triple therapy on elderly patients with mild to moderate osteoporotic vertebral compression fractures (OVCF).

**Methods:**

A total of 114 cases of elderly patients with mild to moderate osteoporotic vertebral compression fractures were identified as research subjects in our hospital from January 2017 to January 2018, and a total of 136 vertebrae were included. The patients who underwent PVP operation were included as the control group with 67 injured vertebrae, and the patients who underwent PKP operation were included as the experimental group with 69 injured vertebrae.

**Results:**

The operation time and bone cement injection volume of the experimental group were significantly higher than the control group. The visual analog scale (VAS) scores of the two groups at 3 months and 6 months after operation were lower than those before operation, with lower VAS scores observed in the experimental group at 3 months and 6 months after operation. The anterior height of the vertebral body in the experimental group was higher than that of the control group. The experimental group outperformed the control group in the incidence of postoperative complications. The postoperative Oswestry dysfunction index (ODI) scores of the two groups were lower before the operation, in which the experimental group had lower scores than the control group (*P* < 0.05).

**Conclusion:**

PVP and PKP combined with postoperative triple therapy can achieve a promising analgesic effect. PKP has a higher volume of bone cement injection volume, and a lower incidence of complications, which gives rise to a better vertebral body recovery height than that of PVP, with rapid postoperative body function recovery and good quality of life.

## Introduction

Osteoporotic vertebral compression fracture (OVCF) is common in the elderly population, and age is an independent risk factor for its onset. It is mainly attributed to the loss of calcium, declined endocrine regulation, or the use of certain drugs in elderly patients, resulting in spinal fracture injury, with symptoms such as long-term back soreness, ligament injury, and spinal deformity (1–3). With the aging population in our country, the incidence rate of the disease is on the rise. Delayed treatment may predispose to a cascade of complications, which will aggravate the disease, seriously compromise the prognosis and quality of life of patients, and even endanger the life of patients (1, 4–6). Zoledronic acid injection is a nitrogen-containing bisphosphate compound that mainly acts on human bones to inhibit bone resorption through the inhibition of osteoclasts. Calcium supplementation after drug administration is considered extremely critical, especially within 10 days after drug administration. Calcium is the most predominant component of bone minerals, and vitamin D promotes the activity of bone cells and thus facilitates calcium absorption. The National Osteoporosis Foundation (NOF) recommended the use of calcium supplements with vitamin D for the prevention of osteoporosis in 2000 (4). In this study, a triple dose of oral osteopontin gel pills and oral calcitrate D was used for treatment. Accordingly, 114 patients with moderate OVCF admitted to our hospital from January 2017 to January 2018 were identified as research subjects, and the therapeutic effects of PVP and PKP combined with triple drugs on elderly patients with mild and moderate OVCF were further analyzed.

## Data and Methods

### General Data

The control group had 56 patients who underwent PVP (67 injured vertebrae), including 32 men and 24 women, aged 56–84 years old, with an average age of (63.5 ± 3.3) years, 37 lumbar vertebrae, and 30 thoracic vertebrae. The experimental group had 58 patients treated with PKP (69 injured vertebrae), including 34 men and 24 women, aged 55–85 years old, with an average age of (64.7 ± 3.2) years old, 40 lumbar vertebrae, and 29 thoracic vertebrae.

### Inclusion Criteria

The inclusion criteria included: (1) Patients who met diagnostic criteria of OVCF; (2) Bone mineral density examination showed osteoporosis in different degrees, and preoperative CT showed that the posterior wall of the vertebral body was intact without spinal cord and nerve root injury; (3) Patients with complete medical records; (4) Patients without other surgical contraindication; (5) This study was approved by the hospital ethics committee. Patients and their family members were informed of the purpose and process of this study, received the treatment plan, and signed the informed consent form.

### Exclusion Criteria

It included: (1) Patients complicated with brain, heart, kidney, liver, and other organ and tissue lesions; (2) Fractured vertebral body compresses spinal cord and nerve; (3) Patients who cannot tolerate surgery; (4) Patients with mental and other cognitive impairments or who refused to cooperate in the experiment.

### Methods

All patients underwent X-ray, CT scan, and magnetic resonance imaging (MRI) examination of the spine before operation. The pedicle of the injured vertebra of the patient was accurately located using a C-arm X-ray machine and marked on the body surface. After preoperative skin preparation and draping with the patients in a prone position, local anesthesia was performed at 1.0–1.5 cm next to the pedicle of the vertebra. A percutaneous puncture was carried out at a 15° angle from the upper lateral side of the pedicle of the vertebra to the sagittal plane, with the 10 o'clock direction of the pedicle of the vertebra being the left puncture point, and the 2 o'clock direction being the right puncture point (7). When the puncture needle reached the posterior edge of the vertebral body, the anteroposterior X-ray film of the injured vertebral body was obtained to observe the integrity of the medial wall of the pedicle to prevent dural and nerve injury. Positioned with a C-arm X-ray machine, the puncture needle was continued when it reached the posterior edge of the vertebral body but did not breach the medial wall of the pedicle, with a puncture depth of 1/3 of the anterior vertebral body.

(1) The control group received PVP. After the puncture needle was hallowed, the prepared bone cement was injected into the vertebral body, which is infiltrated along the trabecular space. The injection was discontinued upon bone cement infiltration into the bone cortex by a C-arm X-ray machine, and the puncture needle was withdrawn after the hardening of the bone cement (8). Bone cement leakage was closely monitored during operation. Intraoperative observation of the cement infiltration by C-arm X-ray is required to determine unilateral or bilateral injection. Unilateral injection was performed in the case of cement infiltration beyond the midline of the vertebral body, and bilateral injection was performed in the opposite case. Injection of bone cement was stopped upon bone cement infiltration to the posterior edge of the vertebral body.

(2) The experimental group underwent PKP. The puncture needle was hallowed and then inserted into the bone drill and the channel was expanded forward to 2–3 mm of the forearm of the vertebral body. Thereafter, the probe was used to explore the four walls of the channel to ensure an intact channel, followed by the insertion of the balloon. The contrast agent was injected to dilate the balloon under the observation of a C-arm X-ray machine, and then the pressure was gradually increased. The contrast agent was withdrawn and the balloon was removed after restoring the vertebral height (9). The injection of the prepared bone cement was consistent with that of the control group.

All patients were given triple medication after operation. One tablet of Calcium D (specification: 1.5 g × 30, Wyeth Pharmaceutical Co., Ltd., Guo Yao Zhun Zi H10950029) was administered orally once a day. Calcitriol capsule (specification: 0.25 g × 10, Qingdao Zhengda Haier Pharmaceutical Co., Ltd., Guo Yao Zhun Zi H20030491) was administered orally twice a day, one tablet each time. The medication of both drugs spanned 1 year. An intravenous drip of zoledronic acid (specification: 5 mg, Novartis Pharmaceuticals of Switzerland, Guo Yao Zhun Zi H20090259) was given one time a year, with a drip length no < 15 min. Also 500 ml of saline was supplemented before administration and 100 ml of saline was administered intravenously to the patient after the infusion. The patients are advised to drink more water after the operation.

### Indicators

#### Surgical Indicators

The operation duration, injection volume of bone cement, and hospitalization duration were counted and compared in the two groups.

#### VAS Score

The visual analog scale (VAS) was used to record the postoperative pain of the patients, with a full score of 10 points. Here 0 points indicate no pain, 0–3 points indicate tolerable slight pain, 4–6 points refer to tolerable pain that affects the sleep, 7–10 points mean unbearable pain that results in poor appetite and sleep.

#### Anterior Height of Vertebral Body

The anterior height of vertebral body before and after operation was recorded and compared.

#### Postoperative Complications

The complications of the two groups were recorded after operation, including bone cement leakage, nerve root pain, chronic pain, and adjacent OVCF.

#### Quality of Life Score

The modified Oswestry dysfunction index (ODI) was adopted to evaluate patients' quality of life.

### Statistical Processing

SPSS 20.0 was used for data analysis, and GraphPad Prism 7 was adopted for image rendering. All measurement data were expressed as ($\bar{x}$ ± s) and analyzed using the *t*-test. The counting data were expressed as [*n*(%)] and analyzed using the Chi-square test. *P* < 0.05 was considered statistically significant.

## Results

### Comparison of General Data

The two groups showed no significant differences in the general data (*P* > 0.05, Table 1).

**Table 1:** Comparison of general data of two groups of patients.

**Category**	**Experimental group**	**Control group**	**X^2^/** ***t***	***P***
Gender			0.0255	0.873
Male	34 (58.62)	32 (57.14)		
Female	24 (41.38)	24 (42.86)		
Age (years old)	64.7 ± 3.2	63.5 ± 3.3	1.9712	0.0512
Smoking history			0.0016	0.968
No	35 (60.34)	34 (60.71)		
Yes	23 (39.66)	22 (39.29)		
Drinking history			0.0061	0.938
No	18 (31.03)	17 (30.36)		
Yes	40 (68.97)	39 (69.64)		
Fracture site			0.1044	0.747
Lumbar spine	40 (57.97)	37 (55.22)		
Thoracic vertebra	29 (42.03)	30 (44.78)		

### Comparison of Related Intraoperative Indexes

The operation duration and bone cement injection in the experimental group were significantly higher than in the control group (*P* < 0.001). There was no statistical difference in hospitalization duration between the two groups (*P* > 0.05, Table 2).

**Table 2:** Comparison of intraoperative indexes ($\bar{x}$ ± s).

**Group**	**Number of vertebral bodies**	**Operation duration (min/vertebral body)**	**Injection volume of bone cement (ml/vertebral body)**	**Length of stay (d)**
Control group	67	30.2 ± 6.9	4.4 ± 2.3	5.6 ± 3.2
Experimental group	69	38.4 ± 9.4	6.1 ± 2.2	6.1 ± 3.1
*t*		5.8113	4.4026	0.8469
*P*		0.000	0.000	0.3989

### Comparison of VAS Score

**Figure 1:**
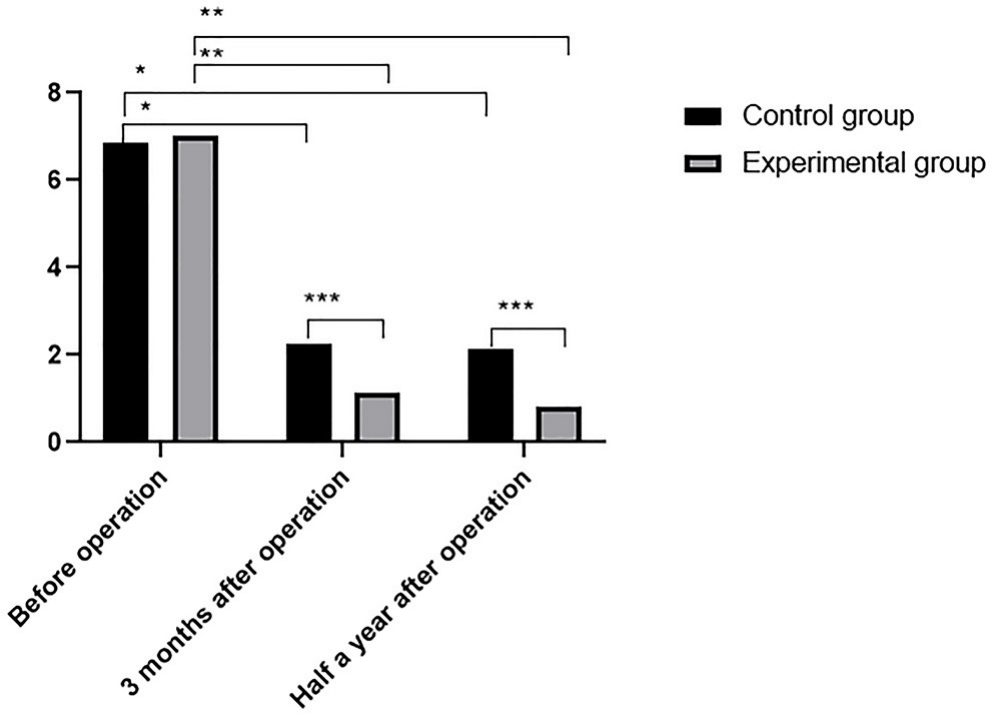
Comparison of VAS scores ($\bar{x}$ ± s). The abscissa indicates the time point of pain evaluation before operation, 3 months postoperatively and 6 months postoperatively, and the ordinate indicates the score. VAS scores of control group before operation, 3 months postoperatively and 6 months postoperatively were (6.85 ± 1.21), (2.25 ± 1.64), and (2.14 ± 1.27), respectively. VAS scores of experimental group before operation, 3 months postoperatively and 6 months postoperatively were (7.03 ± 1.19), (1.12 ± 0.67), and (0.83 ± 0.56), respectively. * Indicates VAS score of control group before operation was significantly different from that 3 months and 6 months postoperatively (*t* = 4.8537, 5.0238, *P* < 0.01); ** indicates VAS score of experimental group before operation was obviously different from that 3 months and 6 months postoperatively (*t* = 32.9581, 35.9021, *P* < 0.01); *** indicates VAS scores of two groups were significantly different at 3 months and 6 months postoperatively (*t* = 4.8460, 7.1678, *P* < 0.01).

Three months and 6 months after surgery, the VAS scores of the two groups decreased markedly, with lower scores observed in the experimental group (Figure 1).

### Comparison of Anterior Height of Vertebral Body

**Figure 2:**
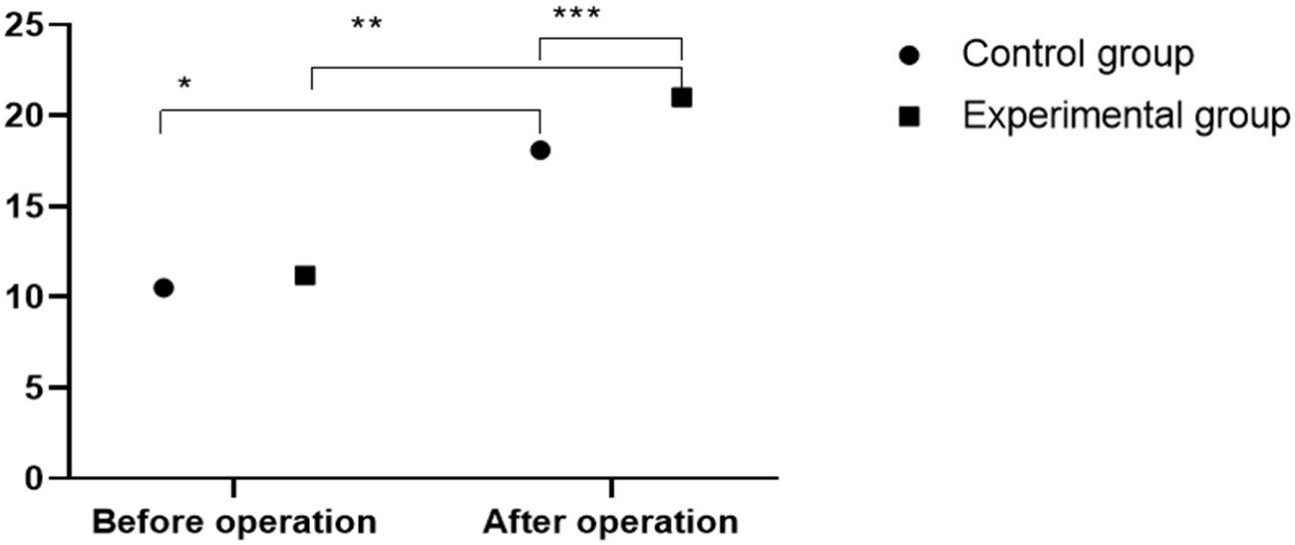
Comparison of anterior height of vertebral body between two groups ($\bar{x}$ ± s). The abscissa indicates preoperation and postoperation, and the ordinate indicates the height of the anterior edge of the vertebral body (mm). The height of anterior vertebral body before and after operation in control group was (10.5 ± 5.1) mm and (18.1 ± 2.4) mm, respectively. The height of anterior vertebral body before and after operation in experimental group was (11.2 ± 4.7) mm and (21.0 ± 6.4) mm, respectively. * indicates the height of anterior vertebral body before and after operation in the control group was significantly different (*t* = 11.0368, *P* < 0.01); ** indicates the height of anterior vertebral body before and after operation in the experimental group was significantly different (*t* = 10.2520, *P* < 0.01); *** indicates there was significant difference in the height of anterior vertebral body between two groups (*t* = 3.5242, *P* = 0.0006).

The postoperative anterior heights of vertebral body in the two groups were remarkably higher than that before operation, in which the results of the experimental group were significantly higher than that of the control group (Figure 2).

### Comparison of Postoperative Complications

The total incidence of postoperative complications in the experimental group was significantly lower than in the control group (*P* < 0.05, Table 3).

**Table 3:** Comparison of the incidence of postoperative complications between the two groups [*n* (%)].

	**Control group (*n* =6 7)**	**Experimental group (*n* = 69)**	***t***	***P***
Bone cement leakage	3 (4.48)	1 (1.45)		
Pain of nerve root	6 (8.96)	2 (2.90)		
Chronic pain	2 (2.99)	0 (0)		
Compression fracture of adjacent vertebral body	0 (0)	0 (0)		
Incidence of complications	11(16.42)	3(4.35)	5.3628	0.021

### Comparison of ODI Scores

**Figure 3:**
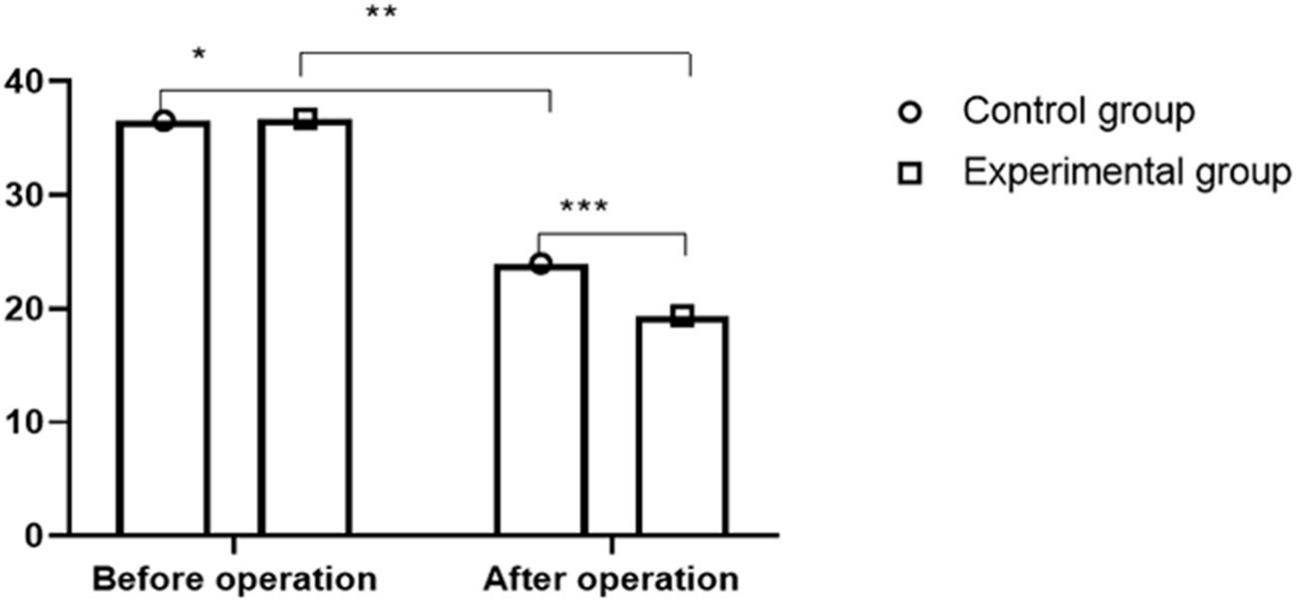
Comparison of ODI scores between two groups ($\bar{x}$ ± s). The abscissa indicates preoperation and postoperation, and the ordinate indicates score. ODI scores of control group before and after operation were (36.54 ±3.37) and (23.97 ± 3.58), respectively. ODI scores of in experimental group before and after operation were (36.72 ± 3.35) and (19.37 ± 3.46), respectively. * indicates ODI score of control patients after operation was significantly lower than that before operation (*t* = 19.1320, *P* < 0.01); ** indicates ODI score of experimental patients was significantly lower than that before operation (*t* = 27.4362, *P* < 0.01); *** indicates ODI scores of two groups were significantly different after operation (*t* = 6. 9765, *P* < 0.01).

After surgery, the ODI scores of the two groups declined significantly, with lower scores obtained in the experimental group than in the control group (*P* < 0.05, Figure 3).

## Discussion

The incidence of OVCF in China has been increasing year by year (10–13). Primary treatment methods for OVCF include nonsurgical treatment, minimally invasive treatment, and surgery. Conservative nonsurgical treatment is unproductive with a long treatment time. Long-term bed rest of patients leads to adverse complications such as falling pneumonia, bedsore, and deep vein thrombosis, which compromises the prognosis and quality of life of patients and greatly increases the economic burden of patients. PVP and PKP, two minimally invasive therapies for osteoporotic compression fractures, contribute to rapid pain relief with small surgical incisions and short postoperative recovery (14–17). Herein, the duration of single vertebral body surgery and bone cement injection in the experimental group were remarkably better than those in the control group, which indicates a more complex PKP procedure and a larger volume of bone cement injection. The VAS scores of the two groups at 3 months and half a year after operation decreased significantly, with lower scores in the experimental group, indicating favorable analgesic effects of the two methods, with a more effective postoperative pain relief of PKP. The height of the anterior edge of the vertebral body in the two groups of patients after operation was remarkably better than the level of another group before operation, in which the experimental group outperformed the control group. The experimental group obtained a lower total incidence of postoperative complications than the control group. The ODI scores of the two groups of patients after operation were significantly lower than those before operation, with better quality of life observed in patients of the experimental group than the control group, which indicated that PKP outperformed PVP in the vertebral body height o and the body function recovery. These results are consistent with the research of Zhou et al. (18), which fully proves that PKP is more effective than PVP in the treatment of moderate osteoporotic vertebral compression fractures.

In summary, patients with mild and moderate OVCF treated with PVP and PKP combined with postoperative triple medication have achieved excellent clinical analgesic effects. In detail, patients with PKP received a large amount of bone cement injection, with a lower incidence of postoperative complications and lower permeability, better recovery of the vertebral body, and quality of life as compared with those who were given PVP.

## Data Availability Statement

The raw data supporting the conclusions of this article will be made available by the authors, without undue reservation.

## Ethics Statement

The studies involving human participants were reviewed and approved by Nantong Hospital of Traditional Chinese Medicine. The patients/participants provided their written informed consent to participate in this study.

## Author Contributions

All authors listed have made a substantial, direct, and intellectual contribution to the work and approved it for publication.

## Funding

This study was supported by the special youth project of clinical medicine funded by Nantong University (Grant No. 2019LQ019).

## Conflict of Interest

The authors declare that the research was conducted in the absence of any commercial or financial relationships that could be construed as a potential conflict of interest.

## Publisher's Note

All claims expressed in this article are solely those of the authors and do not necessarily represent those of their affiliated organizations, or those of the publisher, the editors and the reviewers. Any product that may be evaluated in this article, or claim that may be made by its manufacturer, is not guaranteed or endorsed by the publisher.
